# In situ conversion of defective Treg into SuperTreg cells to treat advanced IPEX-like disorders in mice

**DOI:** 10.1038/s41467-020-15836-2

**Published:** 2020-06-03

**Authors:** Yongqin Li, Yuxin Chen, Shaoshuai Mao, Ravinder Kaundal, Zhengyu Jing, Qin Chen, Xinxin Wang, Jing Xia, Dahai Liu, Jianlong Sun, Haopeng Wang, Tian Chi

**Affiliations:** 1 School of Life Sciences and Technology, Shanghai Tech University, Shanghai, China; 2 CAS Center for Excellence in Molecular Cell Science, Shanghai Institute of Biochemistry and Cell Biology, Chinese Academy of Sciences, Shanghai, China; 3 University of Chinese Academy of Sciences, Shanghai, China; 4 Deptartment Immunobiology, Yale University School of Medicine, New Haven, CT USA; 5 Department of Basic Medicine and Biomedical Engineering, School of Stomatology and Medicine, Foshan University, Foshan, 528000 Guangdong People’s Republic of China; 6 Present Address: Icahn School of Medicine at Mount Sinai, New York, NY USA

**Keywords:** Immunology, Diseases

## Abstract

Mutations disrupting regulatory T (Treg) cell function can cause IPEX and IPEX-related disorders, but whether established disease can be reversed by correcting these mutations is unclear. Treg-specific deletion of the chromatin remodeling factor *Brg1* impairs Treg cell activation and causes fatal autoimmunity in mice. Here, we show with a reversible knockout model that re-expression of *Brg1*, in conjunction with the severe endogenous proinflammatory environment, can convert defective Treg cells into powerful, super-activated Treg cells (SuperTreg cells) that can resolve advanced autoimmunity,  with * Brg1* re-expression in a minor fraction of Treg cells sufficient for the resolution in some cases. SuperTreg cells have enhanced trafficking and regulatory capabilities, but become deactivated as the inflammation subsides, thus avoiding excessive immune suppression. We propose a simple, robust yet safe gene-editing-based therapy for IPEX and IPEX-related disorders that exploits the defective Treg cells and the inflammatory environment pre-existing in the patients.

## Abstract (web summary)

Mutations that affect T_reg_ cell function can cause lethal autoimmunity, but whether correcting these mutations can reverse established disease is unclear. Here, the authors correct T_reg_ cell-specific *Brg1* mutation in a minor fraction of T_reg_ cells, which supercharges these cells to rescue mice from otherwise fatal IPEX-like autoimmunity.

## Introduction

Treg cells are potent suppressors of immune responses^1,2^, and defects in Treg development and/or function cause devastating, fatal autoimmune disorders in humans. Specifically, mutations in *FoxP3*, the master transcription factor in Treg cells, cause the immune dysregulation, polyendocrinopathy, enteropathy, and X‐linked (IPEX) syndrome, with deaths typically ensuing in early infancy or childhood^3–5^, and mutations at other genes important for Treg function cause similar (IPEX-like) disorders^6–8^. Effective treatments for IPEX(-like) disorders remain elusive. However, the revolutionary CRISPR–CAS gene editing technologies are making it possible to treat monogenic diseases such as IPEX(-like) disorders. In particular, in many IPEX(-like) patients, Treg cells are dysfunctional but normal in numbers, which offers an attractive therapeutic target for the gene therapy. In the simplest scheme, viral vectors expressing the editing tools would be injected into the patients, where they would infect the defective Treg cells, correct their mutations, restore Treg functions, and rescue the patients. However, many pitfalls exist. First, Treg cells are difficult to infect. Even if they are successfully infected and mutations corrected, this might be insufficient to (fully) restore Treg functions. Finally, even if all the defective Treg cells in the body could be fully resurrected, it is unclear whether this would be sufficient to resolve the severe ongoing inflammation and rescue the patients, given that the inflammation may have become overwhelming and/or tissue damages irreversible by the time of clinical diagnosis and mutation correction. Obviously, such issues must be carefully addressed before the clinical trials could be launched. One way to address these issues is to use reversible KO mouse models, where a gene important for Treg function is first deleted to recapitulate the IPEX(-like) syndromes, followed by the restoration of gene expression to imitate the clinical intervention. We now report the study on such a model, which involves the chromatin remodeling factor *Brg1*.

*Brg1* is the catalytic subunit of the chromatin remodeling BAF (mSwi/snf) complex^9^, with diverse functions in the immune system^10–15^. We have shown that *Brg1* plays a role in Treg activation^16^. Specifically, the majority of Treg cells under physiological conditions are naive, with little overt suppressor activity. Upon antigen and cytokine stimulation, naive Treg cells become activated and differentiated into effector cells, which migrate to inflamed tissues to efficiently suppress the inflammation^1,17–20^. Importantly, selectively deleting *Brg1* in Treg cells impairs Treg activation, concomitant with the onset of systemic inflammation. As the inflammation progresses, Treg cells become increasingly activated, but the activation levels are unable to catch up with the severity of inflammation, leading ultimately to the death of the KO mice, indicating that *BRG1* acts to facilitate Treg activation^16^. Importantly, the phenotype of our *Brg1* KO mice closely resembles that of the *FoxP3* KO mice, the classic model for IPEX disease, indicating that *Brg1* KO is a valid model of IPEX-like disease, even though *Brg1* is not a known autoimmune disease-associated gene in humans^16^. The *Brg1* KO in this model is irreversible. Therefore, we have now established a reversible *Brg1* KO model, and found that restoring *Brg1* expression in the mice can produce therapeutic effects, with *Brg1* reexpression in only minor fractions (as low as 8%) of Treg cells sufficient for rescuing the mice with slightly less severe phenotypes, suggesting a simple, robust, and safe approach for treating IPEX and IPEX-like diseases.

## Results

### The LOFT strategy for *Brg1*-reversible KO

**Fig. 1:**
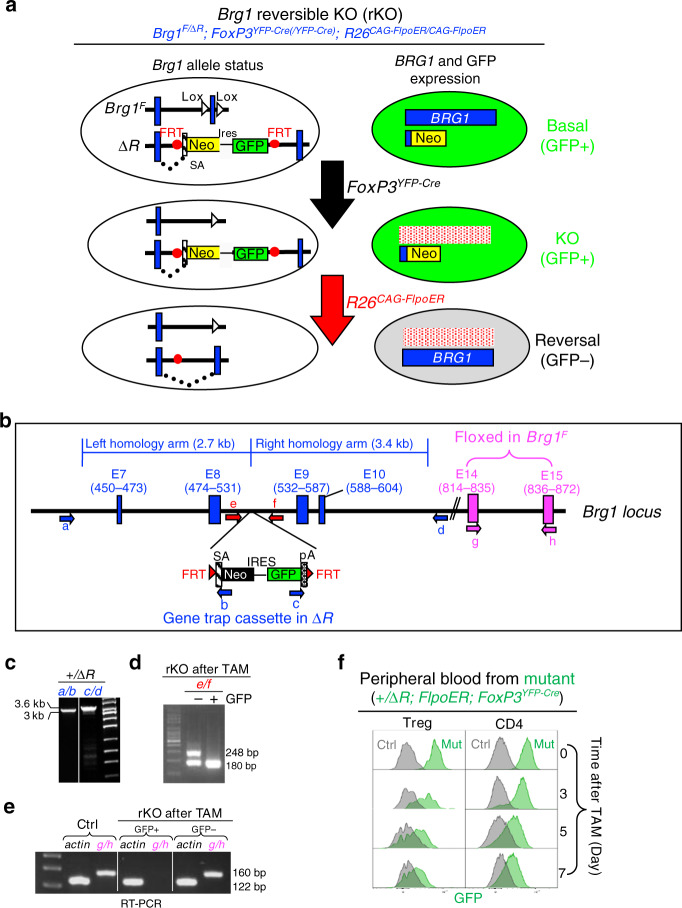
Creation of *Brg1*-reversible KO model using the LOFT method. **a** Strategy for GFP-labeled, Treg-specific reversible *Brg1* knockout. This method requires a conventional *Brg1*-floxed allele (*Brg1*^*F*^) paired with a multifunctional reversible KO (ΔR) allele (top left), and sequential action of Cre and Flpo recombinases (middle and bottom). Depicted is the status of the *Brg1* alleles (left) and the corresponding *BRG1* protein expression patterns (right). Note that Cre was expressed from the endogenous FoxP3 locus located on the X chromosome subject to random inactivation, and so the reversible KO (rKO) mice carried either one or two *FoxP3*^*YFP-Cre*^ alleles depending on the sex. SA splicing acceptor, Neo neomycin-resistance gene, FRT flippase-recognition target (red dot). **b** The *Brg1* alleles. The gene-trap cassette in Δ*R* is inserted after E8 in the *Brg1* locus, and the floxed exons in *Brg1*^*F*^ highlighted in pink. Also depicted are the homology arms used to make the targeting construct for generating Δ*R*, and the PCR primers for genotyping. **c**–**e** Characterization of mouse samples. Shown are the representative results from two biological replicates. The tail from a *Brg1*^*+/*Δ*R*^ mouse was subjected to PCR analysis using primer pairs a/b and c/d (depicted in **b**) to verify successful targeting (**c**); GFP^+^ and GFP^−^ Tregs isolated from TAM-treated rKO mice were analyzed by PCR and RT-PCR to detect the excision of the gene-trap cassette (**d**) and restoration of *Brg1* expression (**e**), respectively. The control mouse in **e** has the same genotype as rKO, except that it carried *Brg1*^*+*^ instead of *Brg1*^Δ*R*^. **f** Kinetics of GFP loss following TAM administration. TAM (full dose) was given via oral gavage, and GFP expression in Tregs and conventional CD4 cells in tail blood monitored by FACS. The control mouse did not carry *Brg1*^Δ*R*^.

Treg-specific *Brg1* deletion followed by conditional restoration of *Brg1* expression was achieved with the LOFT method^21^ that requires a pair of alleles of the target gene (*Brg1* in the current study): a floxed allele (*Brg1*^*F*^) and a reversibly trapped allele that is a null by default, but can be conditionally converted to a wild-type (WT) allele. The latter allele is designated Δ*R*, where R denotes “reversible” (Fig. 1a, top left). The key component of the Δ*R* allele is a gene-trap cassette consisting of the neomycin phosphotransferase (Neo) and Ires-GFP. This cassette was inserted into intron #9 (Fig. 1b), thus capturing the upstream exon #8 (E8) to produce a fusion protein between the N-terminal 531 aa of *BRG1* protein and the neomycin phosphotransferase, the former moiety being inactive, and the latter serving as the selection marker for successfully targeted embryonic stem (ES) cells. In addition, GFP was co-expressed with the fusion protein, which reported the status of Δ*R* allele. The gene-trap cassette was flanked by FLP recombination target (FRT) sites, allowing for conditional cassette excision in the presence of the FLP recombinase. The removal of the gene-trap cassette restores the expression of full-length *BRG1*, concomitant with the loss of GFP expression. Thus, in *Brg1*^*F/*Δ*R*^ mice that also expressed Cre in Treg cells (from the *FoxP3*^*YFP-Cre*^ *allele) and FlpoER* (from the ubiquitous *CAG* promoter inserted into *R26* locus), *Brg1* expression is constitutively eliminated in Treg cells but reinstated upon tamoxifen (TAM) administration, the latter event reported by elimination of GFP fluorescence (Fig. 1a, middle and bottom).

### Characterization of the Δ*R* allele

We inserted the gene-trap cassette into the ES cells using the traditional gene targeting method (Fig. 1b) to generate *Brg1*^*+/*Δ*R*^; *R26*^*CAG-FlpoER*^ mice. Polymerase chain reaction analysis confirmed that the mice carried Δ*R* (Fig. 1c). Following oral gavage of a full dose of TAM (500 µg/g, once daily for 2 consecutive days, termed the “full dose” regimen hereafter), GFP signal in the Treg cells in the peripheral blood decayed gradually, disappearing almost completely on Day 7 after the gavage (Fig. 1f, left), the kinetics being comparable to that in the conventional CD4 cells (Fig. 1f, right). Finally, we bred the reversible KO (rKO) mice by introducing *Brg1*^*F*^ and *FoxP3*^*YFP-Cre*^ into the *Brg1*^*+/*Δ*R*^; *R26*^*CAG-FlpoER*^ mice. As *FoxP3*^*YFP-Cre*^ is located on the X chromosome randomly inactivated in females, the genotypes of rKO mice are gender-specific, being *Brg1*^*F/*Δ*R*^*; FoxP3*^*YFP-Cre*^*; R26*^*CAG-FlpoER/CAG-FlpoER*^ for males and *Brg1*^*F/*Δ*R*^*; FoxP3*^*YFP-Cre/YFP-Cre*^*; R26* ^*CAG-FlpoER/CAG-FlpoER*^ for females. The rKO mice were fed with a low dose of TAM (12 µg/g, once only, termed the “low dose” regimen hereafter) to reverse *Brg1* KO in a fraction of Treg cells. The GFP^+^ and GFP^−^ Treg subsets were then isolated by FACS. As expected, the gene-trap cassette was lost in the GFP^−^ subset (Fig. 1d) concomitant with the emergence of the functional *Brg1* transcript (which contained E14–15; Fig. 1e). These data validated the functionality of the Δ*R* allele.

### *Brg1* reexpression can rescue severely sick rKO mice

**Fig. 2:**
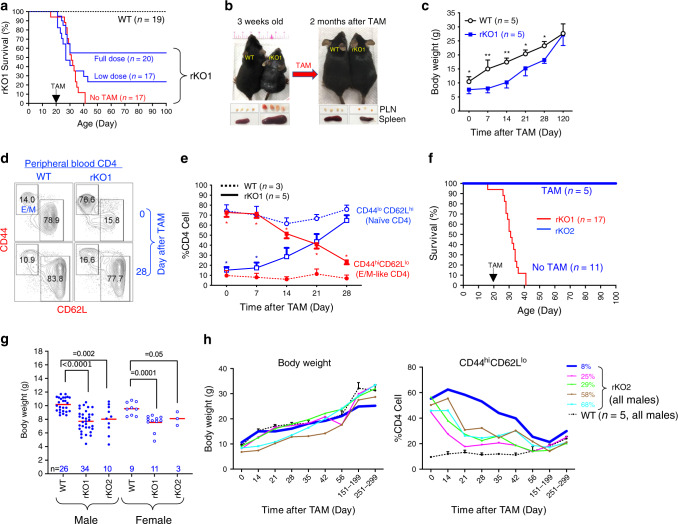
*Brg1* reexpression rescues rKO mice. **a** Survival of rKO1 mice. rKO1 mice were either *Brg1*^*F/*Δ*R*^*; FoxP3*^*YFP-Cre*^*; R26*^*CAG-FlpoER/CAG-FlpoER*^ males or *Brg1*^*F/*Δ*R*^*; FoxP3*^*YFP-Cre/YFP-Cre*^*; R26*^*CAG-FlpoER/CAG-FlpoER*^ females. Littermate controls were of the same genotypes, except that they carried either *Brg1*^*F/*+^ or *Brg1*^Δ*R*/+^ and were thus heterozygous for *Brg1*. For convenience, these controls were labeled “WT” throughout the paper. The rKO1 mice treated with the full dose of TAM lived significantly longer than rKO1 (*p* = 0.01, log-rank test). **b** Representative image of rKO1 mice before and after TAM treatment (full dose). **c** Body weight gain of rKO1 mice following TAM treatment (full dose). * and **P values ≤ 0.05 and 0.001, respectively. **d**, **e** Abundance of effector/memory-like (E/M) and naive CD4 cells in peripheral blood. TAM (full dose) was given to 3-week-old rKO mice (Day 0). Blue and red asterisks denote statistical significance when Naive and E/M CD4 frequencies, respectively, were compared between rKO1 and WT mice. *P values ≤ 0.05 and 0.001. **f** Low-dose TAM regimen fully rescued rKO2 mice (*P* = 0.0007, Student’s *t* test). For comparison, the survival curve for the rKO1 mice (**a**) is also displayed (red line). **g** rKO2 mice were nearly as runted as rKO1. **h** Body weight (left) and E/M CD4 cells (right) of the five TAM-treated rKO2 mice in **f**. The mean values (±SEM) of WT littermates are also plotted (dotted lines). As the rKO mice (bearing five to six alleles) were quite rare, the five mice were born and hence analyzed at different times, except for the last two time points when the mice from different litters were analyzed together (the mouse with 8% reversal analyzed on Days 151 and 251).

The severity of the inflammatory phenotypes was somewhat variable in different rKO mice, and tended to correlate with the frequencies of effector/memory-like (E/M) CD44^hi^ CD62L^lo^ CD4 cells in the peripheral blood. For convenience, we used the frequencies of the E/M CD4 cell at 3 weeks of age to divide the rKO mice into two groups: rKO1 (>65%) and rKO2 (<65%), whose phenotypes are described in Fig. 2a–e and f–h, respectively. Of note, the vast majority (85%) of the rKO mice belonged to the rKO1 category. We found that the rKO1 mice had developed severe inflammatory signs (including skin lesions, lymphoid organ enlargement, and runting) by 3 weeks of age and died before Day 41, the median survival being 31 days (Fig. 2a, red line). To determine the consequences of *Brg1* reexpression in rKO1 mice, mice were given TAM (full dose) around 3 weeks of age, namely ~10 days before the predicted median death date. Remarkably, 55% of the mice (11/20) were rescued from death (Fig. 2a). Gross signs of inflammation disappeared within 2 months after TAM administration (Fig. 2b), and by 120 days, the runted mice had fully caught up in weight and size, revealing striking resilience of the mice (Fig. 2c). To directly examine the kinetics of inflammation resolution, we monitored the proportion of effector/memory-like (E/M, CD44^hi^ CD62L^lo^) and naive-like (Naive, CD44^lo^ CD62L^hi^) CD4 cells within the CD4 cell population in peripheral blood (Fig. 2d). In a 3-week-old rKO1 mouse, the E/M and Naive subset constituted 76% and 16% of the total CD4 population, respectively (as opposed to 14% and 79% in the WT mice, Fig. 2d, top). TAM treatment (full dose) led to pronounced and progressive depletion of the E/M CD4 cells and simultaneous accumulation of the naive CD4 cells, which became apparent within 2 weeks after the treatment (Fig. 2d, e). The reciprocal changes in the abundance of the E/M vs. naive CD4 cells were not due to the conversion of the E/M to naive CD4 cells (Supplementary Fig. 1), and so might instead reflect the changes in their apoptosis/proliferation rates.

We conclude that reversing *Brg1* KO in all of the *Brg1*-deficient Treg cells as late as 10 days before the predicted median death date rescued 55% of the dying mice. However, in clinical settings, it is unfeasible to repair genetic defects in all of the target cells. Therefore, we repeated with the rescue experiment using the low-dose TAM regimen, which resulted in *Brg1* reexpression in variable fractions (10–50%) of Treg cells among different individuals (Supplementary Fig. 2A). Under this condition, 18% (3/17) of the dying rKO1 mice were rescued (Fig. 2a, low dose), with their inflammation resolved and body weight (largely) recovered (Supplementary Fig. 2A, B). *Brg1* reexpression proved more effective in rescuing the rKO2 mice, where inflammation was somewhat less devastating. In the absence of TAM, all but one (11/12) rKO2 mice died before Day 42 and the remaining mice died on Day 67, with the median survival being 38 days, which was only mildly longer than rKO1 mice (Fig. 2f). Furthermore, the rKO2 mice were nearly as runted as rKO1 (Fig. 2g). Thus, rKO2 mice were also very sick. Nevertheless, following the low-dose TAM treatment in 3-week-old mice, which restored *Brg1* expression in 8–68% Treg cells (measured on Day 14 after the treatment; Supplementary Fig. 2D), 100% (5/5) of the rKO2 mice survived (Fig. 2f, blue line), with their body weights catching up and inflammation subsiding over time (Fig. 2h). Remarkably, these changes were observed even in the mouse where *Brg1* expression was restored in only 8% of the Treg cells, despite quite severe inflammation before TAM treatment (Fig. 2h, thick blue line). Note that body weight might never fully catch up, remaining slightly lower than an age- and sex-matched littermate control even on Day 251 after TAM (25.2 vs. 28.8 g). Nevertheless, by Day 251, this mouse seemed to have become otherwise perfectly healthy, devoid of any overt sign of illness such as skin lesions and lethargy (not shown).

Collectively, these data reveal powerful effects of *Brg1* reexpression on the sick mice, with as little as 8% of *Brg1*-reexpressed Treg cells sufficient for the rescue in one case.

### *Brg1* reexpression produces superactivated Treg cells

**Fig. 3:**
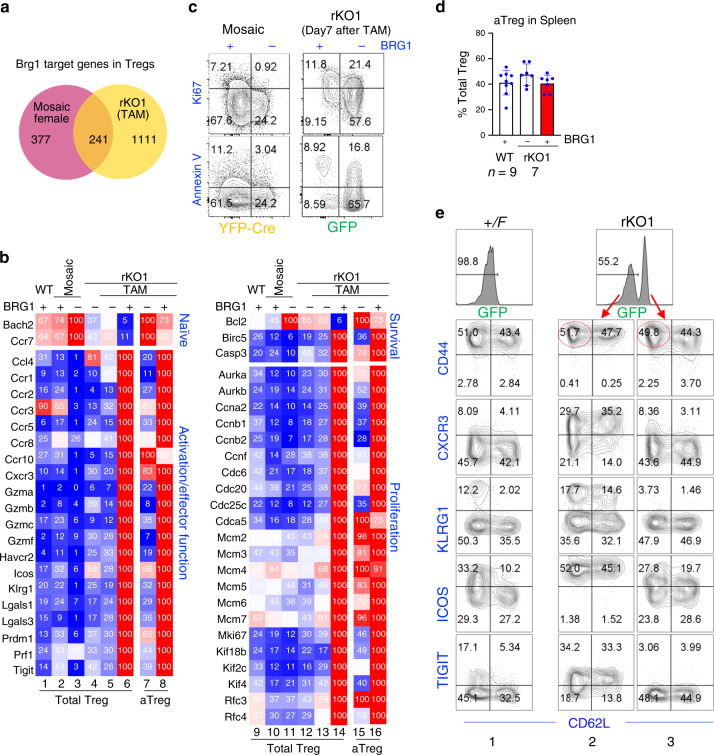
*Brg1* reexpression causes hyperactivation of both rTregs and aTregs. **a** Brg1-target genes from mosaic females and from rKO1 mice on Day 7 after TAM. The target genes were defined as those whose expression was affected ≥2× fold by BRG1. **b** Relative expression of representative Brg1 target genes associated with Treg function (lanes 1–8) or turnover (lanes 9–16). Total Tregs (1–6, 9–14) and aTregs (7–8, 15–16) were isolated and sequenced in two different experiments, and therefore relative expression calculated independently. **c** FACS analysis of proliferation and apoptosis in the Tregs from mosaic females (left) and from the rKO1 mice on Day 7 after TAM (right). The two mice were analyzed at different times. **d**, **e** FACS analysis of aTreg proportion (**d**) and Treg activation marker expression (**e**), showing that in rKO1 mice on Day 7 after TAM, Brg1 reinstated aTreg and rTreg, both expressed activation markers more frequently and, for CXCR3 and ICOS, to higher levels than normal aTregs. The circles in **e** denote the aTregs used for RNA seq in **b**, samples 7–8 and 15–16.

To characterize *Brg1*-reexpressed Treg cells, we treated 3-week-old rKO1 mice with the low dose of TAM and compared gene-expression patterns in *Brg1*-deleted (GFP^+^) vs. *Brg1*-reexpressed (GFP^−^) Treg subsets isolated 7 days after TAM, when the GFP fluorescence in the *Brg1*-reexpressed subset had substantially decayed (Fig. 1f) to enable its clean separation from the GFP^+^ subset. This analysis would reveal the role of *Brg1* in partially activated Treg cells exposed to inflammation. As a control, we addressed the role of *Brg1* in Treg cells under the physiological condition. To this end, we compared *Brg1*-deleted (YFP^+^) and *Brg1*-sufficient (YFP^−^) Treg cells from the healthy, mosaic females (*Brg1*^*F/*Δ*R*^*; FoxP3*^*YFP-Cre/+*^; *R26* ^*CAG-FlpoER/CAG-FlpoER*^, where YFP-Cre was expressed in only half of the Treg cells due to random X inactivation; these mice also carried *R26* ^*CAG-FlpoER/CAG-FlpoER*^ just as the rKO1 mice in order to control for any potential nonspecific confounding effects of FlpoER expression when comparing DE genes between the two strains). As additional controls, we used Treg cells isolated from WT mice and from rKO1 mice not treated with TAM, the former being *Brg1*-sufficient while the latter *Brg1*-deficient, therefore comparable to *Brg1*-sufficient Treg cells from the mosaic females and the *Brg1*-deficient Treg cells from TAM-treated rKO1 mice, respectively. All the mice were 3–4 weeks old when sacrificed. *Brg1* deletion in the mosaic females and *Brg1* reexpression in rKO1 mice on Day 7 after TAM treatment affected 618 and 1352 genes, respectively, with only 241 genes shared, suggesting divergent roles of *Brg1* under the physiological vs. inflammatory conditions (Fig. 3a; see Supplemental Data for a complete list of these genes; raw data already deposited). *Brg1*-target genes are of diverse functions, a conspicuous group being related to Treg function (Fig. 3b, lanes 1–8). These genes can be divided into two categories: the “naïve genes” that are predominantly expressed in naive Treg cells (*Bach2* and *Ccr7*)^19,22^, and “activation/effector function genes” preferentially expressed in activated/effector Treg cells, including many chemokine receptors (*Ccr1*,2,3,5,8,10 and *Cxcr3*)^23^, *Icos*^19^, *Tigit*^24^, *Klrg1* (ref. ^25^), *Prdm1* (ref. ^26^), and *Gzmb*^27^. In the *Brg1* KO Treg cells within the mosaic females, the “naïve genes” were upregulated, while most of the “activation/effector function genes” repressed, relative to the *Brg1*-sufficient Treg cells in both the mosaic females and the WT mice (lane 3 vs. 1–2), confirming that the direct effect of *Brg1* deletion was to inhibit Treg activation^16^. Interestingly, in the rKO1 mice with severe inflammation, the *Brg1* KO Treg cells were partially/weakly activated, with the “naïve genes” repressed and some of the “activation/effector function genes” (i.e., *Cxcr3, Gzma, Gzmb*, and *Gzmf*) upregulated relative to the *Brg1*-sufficient controls (lane 4 vs. 1–2). These data reinforce the notion that *Brg1* KO impairs Treg activation, which triggers inflammation, leading to a secondary partial/weak Treg activation^16^. As expected, in the rKO1 mice, following the low-dose TAM treatment that restored *Brg1* expression in a subset of Treg cells, the *Brg1*-deficient subset remained mostly unaffected, with the expression pattern comparable to that in the rKO1 mice without TAM treatment (lane 5 vs. 4). In sharp contrast, the *Brg1*-reexpressed Treg subset in these mice became dramatically activated, as revealed by 5–10× repression of naive genes and 2–14× upregulation of all the activation/effector function genes relative to the partially activated, *Brg1*-deleted subset (lane 6 vs. 5). Thus, *Brg1* reexpression in the rKO1 mice led to Treg superactivation, the resultant superactivated Treg cells (“SuperTreg cells”) presumably highly potent. The data also demonstrate that although the *Brg1*-target genes were in general highly divergent in the mosaic (healthy) vs. rKO1 (inflamed) mice (Fig. 3a), the *Brg1*-controlled transcription program underlying Treg activation was conserved between the two distinct conditions, but with a twist: in the rKO1 mice, *BRG1* was able to upregulate the activation markers to much higher levels than in mosaic mice (lane 6 vs. 2), which seemed to reflect (in part) a synthetic effect of cytokine stimulation in the rKO1 mice (see further). Activated Treg cells are more apoptotic and proliferative than naive Treg cells^19^. Indeed, in SuperTreg cells, the pro-survival gene *Bcl2* was repressed, whereas the pro-apoptosis gene *Casp3* and many cell-cycle-promoting genes upregulated when compared with all other Treg types examined (Fig. 3b, lane 14 vs. 9–13). Curiously, the pro-survival *Birc5* was also upregulated in SuperTreg cells, perhaps reflecting a negative feedback effect (Fig. 3b, lane 14). FACS analysis confirmed that SuperTreg cells were more proliferative and apoptotic than the *Brg1*-deleted counterpart (Fig. 3c).

We next used FACS to further characterize marker expression on SuperTreg cells. Treg cells consist of two subsets with distinct functions: the highly suppressive “aTreg” (activated Treg, CD44^hi^ CD62L^lo^) enriched in cells expressing activation markers, and the “rTreg” (resting Treg, CD44^hi^ CD62L^hi^) with the opposite phenotype^28^. We found that in the WT control, aTreg cells constituted ~40% of total Treg cells (Fig. 3d), and that activation markers such as ICOS, TIGIT, KLRG1, and CXCR3, hardly detectable in rTreg cells, were each expressed in a fraction of aTreg cells with varying degrees of overlaps, resulting in highly heterogeneous aTreg population (Fig. 3e, column 1 and Supplementary Fig. 3). In rKO1 mice, *Brg1* reexpression did not increase the aTreg abundance (Fig. 3d), but caused more frequent expression of all the four activation markers among aTreg cells (Fig. 3e, column 2 vs. 3 and Supplementary Fig. 3), indicating the aTreg cells were further activated. Interestingly, marker expression was highly induced in rTreg cells (Fig. 3e, column 2), suggesting that rTreg cells also became hyperactivated. To validate and extend the FACS analysis, we used RNA seq to analyze aTreg cells from rKO1 mice. Indeed, for the most part, the *BRG1*-induced changes in the expression profiles revealed further activation of aTreg cells (Fig. 3b, lanes 7–8 and 15–16), although the fold changes were generally smaller than between GFP^+^ vs. GFP^−^ unseparated Treg cells, apparently because of their “cleaner background” resulting from the presence of rTreg cells.

We conclude that *Brg1* reexpression caused hyperactivation of both aTreg and rTreg cells without changing their relative abundance. Of note, whether such activation of rTreg cells is sufficient to make them as suppressive as true aTreg cells is unclear.

### Treg cell hyperactivation requires cytokine signaling

**Fig. 4:**
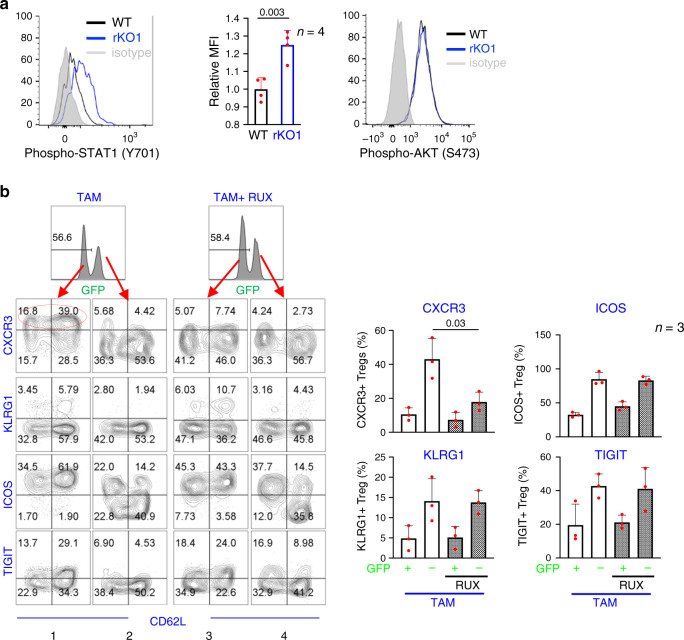
Enhanced Stat1 signaling helps BRG1 drive CXCR3 overexpression. **a** STAT1 and AKT signaling in splenic Tregs. To quantify STAT1 (Y701) phosphorylation, the STAT1 (Y701) MFI for rKO1 Tregs was normalized to the WT controls, the latter set as 1 (middle, where the values represent mean ± SD, *n* = 4). **b** Selective inhibition of CXCR3 expression by Ruxolitinib (RUX). Representative FACS plots were shown at the left (CXCR3 + Tregs in Brg1-reexpessed cells circled) and data displayed at the right. Cells were stained and analyzed as in Fig. 3e. Of note, RUX also appeared to affect CXCR3 in *Brg1*-deleted Tregs, but the effect is hard to quantify because CXCR3 expression was too low.

We have begun to define the mechanism underlying gene hyperactivation in SuperTreg cells, namely, how *BRG1* in the inflamed rKO1 mice could upregulate the activation markers to much higher levels than *BRG1* in the healthy mosaic or WT mice (Fig. 3b, lane 6 vs. 1–2). To address this issue, we used *Cxcr3* as a model. *Cxcr3* marks the Treg subset specialized in suppressing the Th1 response^23^. It is a direct target of *BRG1* (ref. ^16^) and hyperactivated in SuperTreg cells (Fig. 3b). Importantly, *Cxcr3* is induced in response to IFNγ-STAT1 stimulation^23^. Given that *Cxcr3* is subject to joint regulation by *BRG1* and IFNγ-STAT1 pathway, our hypothesis is that in rKO1 mice with severe inflammation, Treg cells experience enhanced IFNγ-STAT1 stimulation, which can conceivably complement *BRG1* to induce strong *Cxcr3* expression. Indeed, in ~3-week-old rKO1 mice with severe inflammation, STAT1 phosphorylation in Treg cells was markedly elevated (Fig. 4a, left and middle). Interestingly, in contrast to IFNγ-STAT1, TCR signaling, as evidenced by AKT S473 phosphorylation, seemed unaltered in these Treg cells (Fig. 4a, histogram at the right).

To verify the causal relationship between STAT1 phosphorylation and CXCR3 expression, we tested the ability of Ruxolitinib, an inhibitor of Stat1/2, to impair CXCR3 induction following *Brg1* reexpression. Mice were pretreated with Ruxolitinib for 2 days before low-dose TAM administration, and then maintained on Ruxolitinib for 7 more days before the analysis. On average, CXCR3 was detectable on 43% of *Brg1-*reexpressing Treg cells, which was indeed markedly reduced (to 18%) by Ruxolitinib (Fig. 4b, column 1 vs. 3, and the top-left graph at the right). This effect of Ruxolitinib was specific, not observed at the three other activation markers (ICOS, TIGIT, and KLRG1) examined (Fig. 4b).

Our data collectively suggest that *Brg1* reexpression acted in conjunction with inflammatory cytokines to convert *Brg1*-deleted Treg cells into hyperactivated SuperTreg cells.

### Enhanced function of SuperTreg cells in vitro and in vivo

**Fig. 5:**
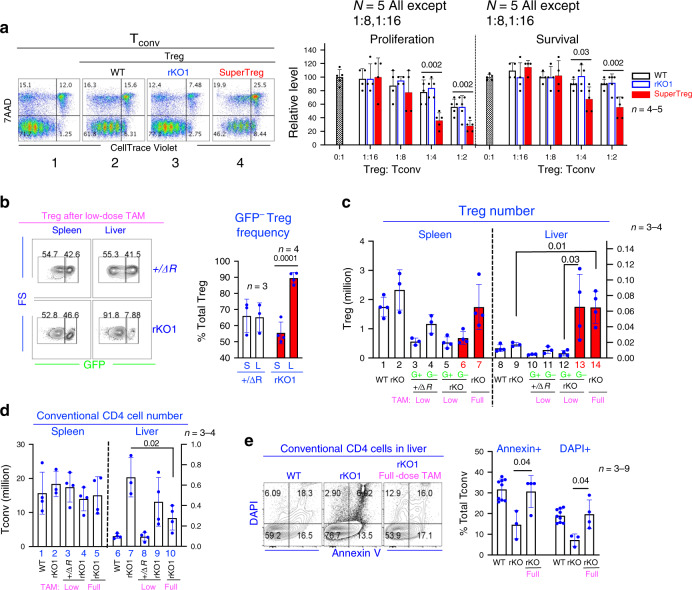
SuperTreg cell function in vitro and in vivo. **a** SuperTreg suppression in vitro. Conventional CD4 cells (Tconv), labeled with CellTracer, were stimulated with APC and anti-CD3 in the presence or absence of Tregs for 5 days before analyzing 7-AAD and CellTracer fluorescence. Representative FACS plots were shown at the left (Treg:Tconv = 1:4). The inverse of CellTracer MFIin total Tconv and the proportions of viable (7-AAD^−^) Tconv were taken as measures of Tconv proliferation and survival, respectively, which are displayed as the percentages of the control (Treg:Tconv = 0:1). *n* = 5 repeats for all the conditions, except 1:16 and 1:8 (*n* = 4); the data are pooled from four independent experiments. **b**, **c** SuperTreg accumulation in the liver. rKO1 mice were treated with TAM and analyzed 7 days later for Treg frequency (**b**) and number (**c**) in the spleen (S) and liver (L). In **b** low-dose TAM was used, leading to GFP loss in only a fraction of Tregs, as shown in the FACS plot and summarized in the bar graph. *+/*Δ*R* controls were *Brg1*^*+/*Δ*R*^*;FoxP3*^*YFP−Cre*^*; R26* ^*CAG-FlpoER*^. “WT” mice included +/Δ*R* and +/*F* mice as mentioned in Fig. 2a legend. G+ and G−, GFP+ and GFP− Treg subsets, respectively. In **c** *n* = 4 for all mice, except *rKO* and +/DR (*n* = 3). **d**, **e** The number (**d**) and death (**e**) of conventional CD4 cells. The bar graph in **e** summarizes total Annexin+ and total DAPI + conventional CD4 cells. Mice were described in **b**, **c** except that five more WT data points were pooled. In **d** *n* = 4 for all mice, except *rKO1* (*n* = 3); in **e** *n* = 9 (*WT*), 3 (*rKO*), or 4 (*rKO* full).

We first evaluate the suppressive function of SuperTreg cells in vitro. To this end, dye-labeled conventional CD4 cells (Tconv) were stimulated with antigen-presentation cells and anti-CD3 for 5 days in the presence or absence of Treg cells before FACS analysis. In the absence of Treg cells, the majority of Tconv had undergone multiple rounds of division as revealed by progressive dye dilution (Fig. 5a, FACS plot 1). WT Treg cells inhibited proliferation in a dose-dependent manner, with the relative proliferation decreased to ~78% and ~56% of the control level at the Treg:Tconv ratios of 1:4 and 1:2, respectively (Fig. 5a, FACS plot 2 and bar graph at the right), which is comparable to that measured by [3H]thymidine incorporation we and others previously reported^16,25,29^. The *Brg1*-deleted Treg cells from rKO1 mice were of similar potency to WT Treg cells (FACS plot 3), whereas importantly, SuperTreg cells were ~2× as potent, reducing the proliferation to ~36% and ~28% at the Treg:Tconv ratios of 1:4 and 1:2, respectively (FACS plot 4 and bar graph at the right). SuperTreg cells were also more potent in killing, reducing the survival rates of Tconv to ~68% and ~56% at the Treg:Tconv ratios of 1:4 and 1:2, respectively, compared with >90% observed in the presence of WT and *Brg1*-deleted Treg cells under these conditions (Fig. 5a). It is noteworthy that the apparent killing efficiency of WT Treg cells in our assay was lower than that described in a previous study, where at the end of a 3-day incubation, WT Treg cells reduced Tconv survival rate by ~2× at the Treg:Tconv ratios of 1:2 and 1:1 (ref.^27^). This discrepancy arose perhaps because we incubated the cells for 5 days (instead of 3) before the analysis, which would lead to more extensive proliferation of the surviving Tconv and thus more severe dilution of the dead cells. The dead cells were also more likely to become undetectable (due to disintegration) by Day 5, further decreasing the ostensible Treg killing efficiencies in our assay. In any case, it is clear that SuperTreg cells displayed enhanced suppressive function in vitro. However, the enhancement was quite moderate, which could hardly explain the dramatic effect of SuperTreg cells in vivo. Indeed, among the *Brg1*-target genes listed in Fig. 3b, only Granzyme B has been shown to contribute to in vitro suppression (and perforin unexpectedly dispensable^27^), while multiple other target genes are apparently involved in Treg function only in vivo. These genes include the chemokine receptors mentioned above (*Ccr1*,2,3,5,8,1 and *Cxcr3*), whose upregulation predicts that SuperTreg cells should migrate to inflamed tissues more efficiently than the *Brg1*-deleted counterpart. To prove the concept, we examined SuperTreg abundance in the liver from the rKO1 mice, using as a control the +/Δ*R* mice carrying a copy of the WT *Brg1* allele in combination with the reversible KO allele. The mice were treated with low-dose TAM, which reversed the KO (marked by loss of GFP expression) in a fraction of Treg cells in both strains. In the rKO1 mice, the reversal would convert the *Brg1*-deleted Treg cells to SuperTreg cells highly expressing the chemokine receptors, but the reversal would be inconsequential in the control mice already carrying the WT allele. We found that in the control +/Δ*R* mice, on Day 7 following TAM treatment, the frequencies of GFP^−^ Treg in the liver and spleen were comparable (~65% of total Treg cells on average in both cases; Fig. 5b, c, bar 3–4 and 10–11). In the rKO1 mice, the GFP^−^ Treg cells (namely SuperTreg cells) were of similar abundance to the GFP^+^ subset in the spleen (Fig. 5b, c, bar 5 vs. 6) but 10-fold more abundant than the latter in the liver (Fig. 5b) where the SuperTreg cells totaled ~65,000 (compared with ~6000 for the GFP^+^ subset; Fig. 5c, bar 13 vs. 12). We also treated the rKO1 mice with full-dose TAM, which converted all *Brg1*-deleted Treg cells into SuperTreg cells. In these mice, SuperTreg cells also accumulated in the liver (Fig. 5c, bar 14), significantly outnumbering (by 3.8-fold) those in the control rKO1 mice lacking TAM treatment (cellularity ~65,000 vs. ~17,000, Fig. 5c, bar 14 vs. 9). The SuperTreg cells in the rKO1 mice treated with low-dose TAM also outnumbered the control (bar 13 vs. 9), but the difference is insignificant due to large individual variations in the SuperTreg cellularity in these particular three rKO1 mice analyzed. As expected, the hepatic SuperTreg cells were as hyperactivated as the splenic counterpart (Supplementary Fig. 4A). To determine the functional consequence of the hepatic accumulation of SuperTreg cells, we focused on the conventional CD4 cells in the liver, as these cells are the key effector in the disease pathogenesis in the *scurfy* and, by inference, the rKO1 mice^30^. In rKO1 mice, the CD4 cells had infiltrated the liver, totaling ~0.7 million (compared with ~0.1 million in the WT mice; Fig. 5d, bars 6–7), which was reduced ~2.6-fold (to ~0.27 million) within 7 days following full-dose TAM treatment (bar 10); the same trend was observed following low-dose TAM treatment (bar 9). The CD4 cell depletion concomitant with SuperTreg accumulation led to a dramatic (~10-fold) increase in the Treg:Tcon ratio in the rKO1 mice following full-dose TAM treatment. To explore the mechanism of CD4 cell loss, we examined its apoptosis. Interestingly, there were ~2-fold fewer apoptotic/dead cells in rKO1 mice than in the WT mice, and importantly, TAM caused ~2-fold increases in the apoptosis (Fig. 5e). These data strongly suggest that following TAM treatment, SuperTreg cells emerging in the spleen and lymph nodes flocked to inflamed tissues to overwhelm inflammation (in part) by killing conventional CD4 cells. Of note, beside chemokine receptors and granzymes/perforin, some other *Brg1*-target genes could also contribute to SuperTreg potency in vivo, including ICOS important for the stability of FoxP3 expression^31^. Thus, SuperTreg cells may use multiple mechanisms to resolve the inflammation.

### The fate of SuperTreg cells in vivo

We have followed SuperTreg cells in the five TAM-treated rKO2 mice (Fig. 2h); these mice, treated with the low-dose TAM regimen, harbored both GFP^−^ and GFP^+^ Treg subsets, the former being SuperTreg cells, while the latter serving as an internal control for FACS analysis. Peripheral blood was drawn and the four Treg markers (KLRG1, ICOS, TIGIT, and CXCR3) monitored over time.

**Fig. 6:**
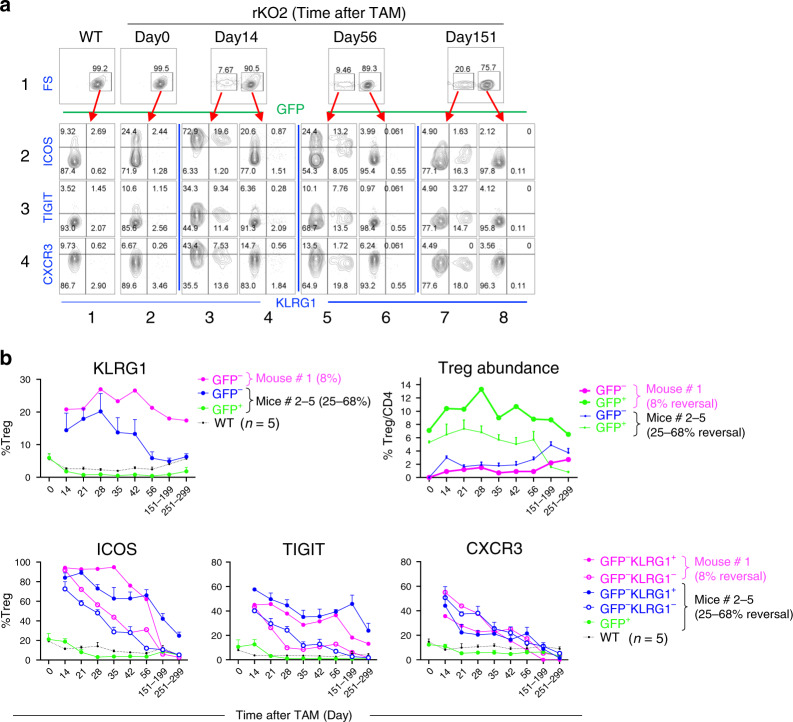
Fate of SuperTreg cells in vivo. **a** Peripheral blood cells from the rKO2 mouse with 8% GFP^−^ Tregs (Fig. 2h, thick blue line) were stained with a mixture of CD4, KLRG1, ICOS, TIGIT, and CXCR3 antibodies for the analysis of ICOS, TIGIT, and CXCR3 expression in KLRG1^+^ and KLRG1^−^ subsets (rows 2–4) within the GFP^−^ (columns 3, 5, and 7) and GFP^+^ (columns 1, 2, 4, 6, and 8) Treg populations. **b** Summary of the FACS results for the mouse analyzed in **a** (mouse #1), together with the four other mice in Fig. 2h (#2–5). For clarity, only the GFP^−^ subset is displayed for mouse #1 in all the plots, except the top-right plot, and only mean ± SEM is displayed for Mice #2–5. As the rKO mice (bearing five to six alleles) were quite rare, the five mice were born and hence analyzed at different times, except for the last two time points when the mice from different litters were analyzed together (the mouse with 8% reversal analyzed on Days 151 and 251).

In the five mice, SuperTreg cells comprised 8–68% of total Treg cells in the blood on Day 14 after TAM (Fig. 2h). We were especially intrigued in the mouse harboring the least (8%) amount of SuperTreg cells (thick blue line, Fig. 2h). In this particular mouse, the KLRG1^+^ Treg subset, barely detectable within *Brg1*^−^-deleted (GFP^+^) Treg subset, accounted for as much as 21% of the SuperTreg population on Day 14 after TAM, which remained elevated thereafter, presumably reflecting the persistence of a certain degree of inflammation (Fig. 6a, rows 2–4; Fig. 6b, top left, pink line). By Day 14 after TAM, ICOS had been dramatically induced in SuperTreg cells, being expressed on (almost) all KLRG1^+^ and KLRG1^−^ subsets (as opposed to 21% in *Brg1*-deleted Treg cells; Fig. 6a, row 2, column 4). Interestingly, in contrast to KLRG1, ICOS expression in SuperTreg cells (and in *Brg1*-deleted Treg cells) declined over time to the baseline by Day 251 after TAM, occurring faster in the KLRG1^−^ subset (Fig. 6a, row 2, columns 3–8; Fig. 6b, bottom left), suggesting (partial) resolution of inflammation. Indeed, TIGIT and CXCR3, also induced on SuperTreg cells (albeit to less extents than ICOS), had similarly declined to (near) basal levels by Day 251 (Fig. 6a, rows 3–4; Fig. 6b, bottom), as was the abundance of the E/M CD4 cells in the peripheral blood (Fig. 2h, right). Of note, on Day 151 after TAM, the frequency of GFP^−^ Treg subset within the Treg population was markedly increased (to 20.6% from 9.5% on Day 56, Fig. 6a, row 1). To determine whether this increase was due to the accumulation of the GFP^−^ Treg subset and/or depletion of the GFP^+^ Treg subset, we examined the abundance of the two Treg subsets relative to that of conventional CD4 cells, finding that the increased frequency of the GFP^−^ subset was due to its accumulation, because the abundance of the GFP^+^ subset remained constant as compared with Day 56 (Fig. 6b, right, heavy lines). Interestingly, by Day 251, GFP^+^ subset had become partially depleted, while the GFP^−^ subset further accumulated (Fig. 6b, right, heavy lines, last time point). Further studies are needed to clarify the mechanism underlying these phenomena.

In the remaining four rKO2 mice (#2–4), where *Brg1* was reexpressed in more (25–68%) Treg cells (Fig. 2h), the E/M CD4 cells were depleted far more rapidly (Fig. 2h, bottom), and all the activation markers (including KLRG1) decayed over time (Fig. 6b), consistent with more effective resolution of inflammation. The reciprocal changes in the abundance of the GFP^−^ vs. GFP^+^ Treg subsets were also observed (Fig. 6b, top right, thin lines). Finally, we also followed the fate of the three rKO1 mice treated with the low-dose TAM regimen, with similar findings (Supplementary Fig. 2C). We conclude that SuperTreg cells tended to lose the hyperactivated phenotype as the inflammation subsided, suggesting that the inflammatory environment was essential for maintaining Treg hyperactivation.

## Discussion

We propose the following model based on the current and the previous work^16^ (Supplementary Fig. 5). In WT mice, when antigens activate conventional T cells, Treg cells also get activated to restrict the immune response. In rKO mice, *Brg1* KO impairs Treg activation, leading to the onset of inflammation. As the inflammation intensifies, Treg cells get partially activated (partly) by inflammatory stimuli such as IFNγ, but this is insufficient to stop the ongoing inflammation (dotted line). Importantly, at this point, BRG1 reexpression (upon TAM treatment) can act in conjunction with the inflammatory stimuli to convert the defective Treg cells into hyperactivated SuperTreg population, in which not only the aTreg cells but also rTreg cells express higher levels of activation markers than WT aTreg cells (let alone WT rTreg cells). SuperTreg cells are highly potent, capable of resolving severe inflammation (solid line) and rescuing the dying mice, with a very small number (8% of total Treg cells) sometimes sufficient for the rescue (not depicted). The potency of SuperTreg cells results at least in part from their enhanced trafficking and suppression abilities. Importantly, as the inflammation subsides, SuperTreg cells reverse activation-induced changes, thus avoiding excessive/persistent immune suppression (not depicted). Of note, TAM treatment should also lead to *Brg1* reexpression in the Treg precursors in the thymus and bone marrow, and the nascent, *Brg1*-sufficient Treg cells might also contribute to the resolution of inflammation. However, this contribution might be minimal, given the low rate of T-cell production in adult mice, especially in the sick mice where the thymi were profoundly atrophic and DP virtually absent presumably as a result of inflammatory stress (Supplementary Fig. 6). It is also noteworthy that while SuperTreg cells were highly potent in rKO mice, they could not outperform WT Treg cells in preventing the weight loss in an IBD model established by injecting conventional CD4 cells into the Rag KO mice (Supplementary Fig. 4C). This is perhaps because the colitis model is established in the lymphopenic mice and different in many ways from rKO mice.

Despite years of research, treatment options for the IPEX(-like) disorders are limited mainly to immunosuppressive drugs and allogeneic hematopoietic stem cell transplantation (HSCT). Immunosuppressive therapy is beneficial only temporarily, as it fails to prevent disease progression in most patients, with the overall survival rate being only 65% at 24 years of age^7^. HSCT does not improve the survival rate, and furthermore, some patients cannot undergo HSCT due to limited donor availability or because their clinical manifestations are not severe enough to justify HSCT^7,32^.

An exciting alternative method to treat IPEX(-like) disorders is gene therapy^33^, and several approaches have been proposed, which entail ex vivo genetic manipulation of HSCs or conventional CD4 cells isolated from the patients, followed by transfer of the modified cells back into the patients^34–36^. Our study suggests a much simpler but powerful and perhaps safer approach that would bypass the ex vivo procedures. Specifically, in some IPEX(-like) patients, the Treg cells are dysfunctional but normal in numbers^33^. For such patients, viral vectors expressing gene editors might be systemically delivered, which would correct the Treg mutations and potentially convert them into SuperTreg cells. This conversion is plausible if the mutations compromise Treg activation in a reversible manner as in the case of *Brg1* KO. Alternatively, the mutations might not affect Treg activation, but block some other aspects of Treg function. In this case, the defective Treg cells should already be hyperactivated prior to gene therapy, and if the particular Treg defects are (partially) reversible, then repairing the mutations might suffice to convert the Treg cells into SuperTreg cells. This strategy would not only be simple and powerful, but also safe in the sense that it is free from the risk of “drug over dose” (SuperTreg cells get deactivated as the inflammation subsides, thus avoiding excessive immune suppression), in contrast to strategies like infusion of exogeneous Treg cells. Importantly, a lentiviral vector has been developed that can transduce 7% of human CD4 cells in mice following a single i.p injection^37,38^. As conversion of only 8% of defective Treg cells into SuperTreg cells could suffice for the rescue in our mouse model, some therapeutic benefits could perhaps already emerge even when only 7% of Treg cells in the patients are transduced. Finally, a recent elegant study demonstrates that *FoxP3*-deficient Treg cells could be functionally restored by manipulating metabolic pathways^39^. Our strategy is of course not mutually exclusive with such treatments.

## Methods

### Mice

*Brg1*^*ΔR*^ allele was generated using traditional gene targeting strategy as described for the *Baf57*^*ΔR*^ allele^21^, except that the homology arms in the *Baf57*^*ΔR*^ targeting construct were replaced with the sequences from the *Brg1* locus (Fig. 1a). The rKO mice were then created by introducing *Brg*^*F*^ (ref. ^40^), *R26*^*CAG-FlpoER*^ (ref. ^41^) (JAX 19016), and *FoxP3*^*YFP-Cre*^ (ref. ^42^) (JAX 016959) into the *Brg1*^Δ*R/+*^ mice. Before being bred to generate the rKO mice, the *Brg1*^*ΔR*^ mice (created on mixed C57BL/6;129 Sv background) had been backcrossed for 3 generations to C57BL/6, and all other strains for >8 generations. All animals were housed under the SPF condition at ~20 °C with ~50% humidity. Male and female mice of different ages (20–299 days) were used in this study as indicated in the figure legends.

### Tamoxifen treatment

For full-dose regimen, 50 mg of tamoxifen (TAM, Sigma Aldrich) was added to 900 µl of corn oil plus 100 µl of 100% ethanol (50 mg/ml final concentration), and dissolved by incubation at 55 °C for 30 min. The solution can be stored at −20 °C. The drug was delivered (typically into 3-week-old mice) via oral gavage at 10 µl/g body weight, once a day for 2 consecutive days. Low-dose regimen was identical, except that the drug was at lower concentration (1.25 mg/ml) and delivered by a single gavage, translating to 40 × less TAM as compared with the full dose regimen. While the full-dose regimen invariably caused complete deletion of the gene-trap cassette, the low-dose regimen produced variable, highly unpredictable deletion, with the efficiencies ranging from 7 to 70% in different individuals.

### Ruxolitinib treatment

Three-week-old mice were given ruxolitinib (LC LABS, 60 mg/kg in PBS/0.1% Tween 20) via oral gavage once daily for 2 days before low-dose TAM administration. The mice were maintained on ruxolitinib for 7 more days before analysis.

### Flow cytometry

Lymphocytes were isolated from the spleen and lymph node by crushing the tissues over a 20-μm cell strainer (Becton Dickinson). To isolate intrahepatic lymphocytes, the liver in anesthetized mice was perfused with PBS via the portal vein until the liver was opaque, and then pressed through a 70-μm cell strainer (Becton Dickinson). The total liver cells were then resuspended in a 40% isotonic Percoll solution (Amersham Pharmacia Biotech) underlaid with a 60% isotonic Percoll solution. After centrifugation for 20 min at 900*g*, lymphocytes were recovered at the 40/60% interface and washed once with PBS before use. Cells were stained with antibodies and analyzed using FACS Fortessa (BD Biosciences) and sorted using FACSAria (BD Biosciences). Phospho-STAT1 and phospho-AKT in splenic Treg cells were detected using the following cocktail and the Transcription Factor Buffer Set (BD Pharmingen, 562574): CD4-BV650 or CD4-APC (RM4-5, Biolegend), FoxP3-Percp5.5 (R16-715, BD), Stat1 (pY701)-PE-Texas Red (4A, BD), and AKT (pS473)-BV421 (M89-61, BD). To minimize sample-to-sample variation of phospho-STAT1 and phospho-AKT signals, WT and rKO1 splenocytes were stained with CD4-BV650 and CD4-APC, respectively, before the cells were pooled and stained with the remaining antibodies. The cells in Fig. 4 were analyzed with the following cocktail: CD4-BV650 (Biolegend), CD25-BV605 (PC61, Biolegend), ICOS-PE (15F9, Biolegend), CXCR3-APC (Cxcr3-173, eBiosciences), KLRG1-BV421 (2F1, BD), and TIGIT-APC-R700 (1G9, BD). Other antibodies used include CD44-PE-Cy7 (IM7, Biolegend), CD62L-APC (MEL-14, Biolegend), and KI-67-PE (16A8, BD). FACS data were analyzed by Flowjo 10.6.1.

### Gene-expression profiling by RNA seq

Lymphocytes from lymph nodes and spleens from 3- to 4-week-old mice were first magnetically depleted of non-CD4 cells before electronic sorting of Treg cells (CD4^+^CD25^+^YFP^+^) or aTreg cells (CD4^+^CD25^+^YFP^+^CD44^hi^ CD62L^lo^). Total RNA was isolated from 0.1 million Treg cells using RNAprep Pure Micro Kit (TIANGEN), and cDNA synthesized from mRNA using SMART-Seq® v4 Ultra™ Low Input RNA Kit (Clontech). The library was then constructed and sequenced on IlluminaHiSeq platform with the PE150 strategy, which yielded 25–60 million reads per sample. To identify differentially expressed (DE) genes between the *Brg1*^+^ and *Brg1*^*−*^ Treg subsets in mosaic and TAM-treated rKO1 mice, the count data were TMM normalized, the genes <5 cpm for both subsets filtered out, and the *p* values adjusted by the Benjamini and Hochberg method. DE genes are defined as those with absolute fold changes ≥2 and *padj* < 0.05.

### In vitro suppression assay

Conventional CD4 and Treg cells were isolated from PLN and spleens from 3- to 4-week-old mice. CD4^+^ cells were first enriched using Mouse CD4 T Cell Isolation Kit (Biolegend) before electronic sorting. *Brg1* KO Treg and SuperTreg cells were isolated from rKO1 mice before and 7 days after TAM, respectively, while *Brg1*-sufficient littermates (*Brg1*^*F/+*^*; FoxP3*^*YFP-Cre(/YFP-Cre)*^*; R26*^*CAG-FlpoER/CAG-FlpoER*^) used as the source of conventional CD4 cells (CD4^+^CD25^−^YFP^−^) and WT Treg cells (CD4^+^CD25^+^YFP^+^). The purity of conventional CD4 and Treg cells exceeded 95% and 90%, respectively. To assess Treg function, conventional CD4 cells (5 × 10^4^) were labeled with CellTrace Violet (GIBCO) and stimulated with *Rag1*^*−/−*^ splenocytes (5 × 10^4^) plus 1 µg/ml anti-CD3e in the presence of indicated numbers of Treg cells. Five days later, the cells were stained with 7^−^ AAD and anti-CD4-APC before flow cytometrical analysis of proliferation and survival of the conventional CD4 cells. *Statistics*: Unless stated otherwise, an unpaired Student’s *t* test (two tailed) was performed to test for statistical significance, with the values representing mean ± SD (Figs. 2c, 3d, 4a, b, 5a–e) or mean ± SEM (all other cases). Where indicated, * and ** signify *p* values ≤ 0.05 and 0.001, respectively. All statistics were calculated using Prism 8 (GraphPad).

### Study approval

All mouse studies were approved by the IACUC at the Shanghai Institute of Biochemistry and Cell Biology, Chinese Academy of Sciences, and conducted in an AAALAC-accredited facility in compliance with the relevant regulations.

## Supplementary information

Supplementary Information

## Data Availability

RNA-seq data have been deposited in the Gene Expression Omnibus and are available under the accession code GSE132562. Raw data for figures are provided in the Source Data file. All other data are available in the paper and its Supplementary files or from the author upon request.

## Source Data

Source Data
