# Author Correction: In situ conversion of defective Treg into SuperTreg cells to treat advanced IPEX-like disorders in mice

**DOI:** 10.1038/s41467-021-22629-8

**Published:** 2021-04-08

**Authors:** Yongqin Li, Yuxin Chen, Shaoshuai Mao, Ravinder Kaundal, Zhengyu Jing, Qin Chen, Xinxin Wang, Jing Xia, Dahai Liu, Jianlong Sun, Haopeng Wang, Tian Chi

**Affiliations:** 1 School of Life Sciences and Technology, Shanghai Tech University, Shanghai, China; 2 CAS Center for Excellence in Molecular Cell Science, Shanghai Institute of Biochemistry and Cell Biology, Chinese Academy of Sciences, Shanghai, China; 3 University of Chinese Academy of Sciences, Shanghai, China; 4 Deptartment Immunobiology, Yale University School of Medicine, New Haven, CT USA; 5 Department of Basic Medicine and Biomedical Engineering, School of Stomatology and Medicine, Foshan University, Foshan, 528000 Guangdong, PR of China; 6 Present Address: Icahn School of Medicine at Mount Sinai, New York, NY USA

**Keywords:** Immunology, Diseases

Correction to: *Nature Communications* 10.1038/s41467-020-15836-2, published online 03 June 2020.

The original version of this Article omitted the following from the Acknowledgements:

We thank the Molecular and Cell Biology Core Facility (MCBCF) at the School of Life Science and Technology, ShanghaiTech University, for providing technical support.

This has now been corrected in both the PDF and HTML versions of the Article.

