# Supplementary material for: In situ conversion of defective Treg into SuperTreg cells to treat advanced IPEX-like disorders in mice
Source: Nat Commun. 2020 Jun 3;11:2781. doi: 10.1038/s41467-020-15836-2 (PMC7271236; doi:10.1038/s41467-020-15836-2)
Supplement: Supplementary file 1 — Supplementary Information [file 41467_2020_15836_MOESM1_ESM.pdf]

# Supplementary Information

Li et al.

A reversible gene KO strategy reveals therapeutic potentials of defective Treg cells

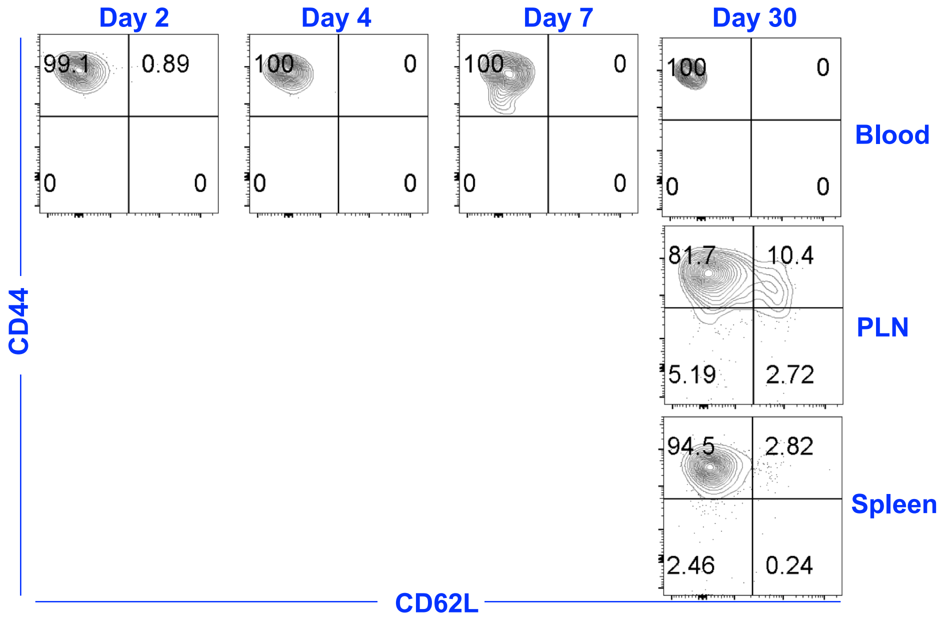


**Supplementary Figure 1**. E/M CD4 did not change to naïve CD4 *in vivo*. E/M CD4 cells were isolated from rKO1 mice, labeled with Celltracer Violet before adoptive transfer into an rKO1 mouse just treated with the full dose of TAM. The labeled E/M cells remained $CD44^{hi}CD62L^{lo}$ in the blood, and some of the them in the LN and spleen expressed CD62L one month after the transfer. However, none of them was converted to $CD44^{lo}CD62L^{hi}$ naïve CD4 cells.

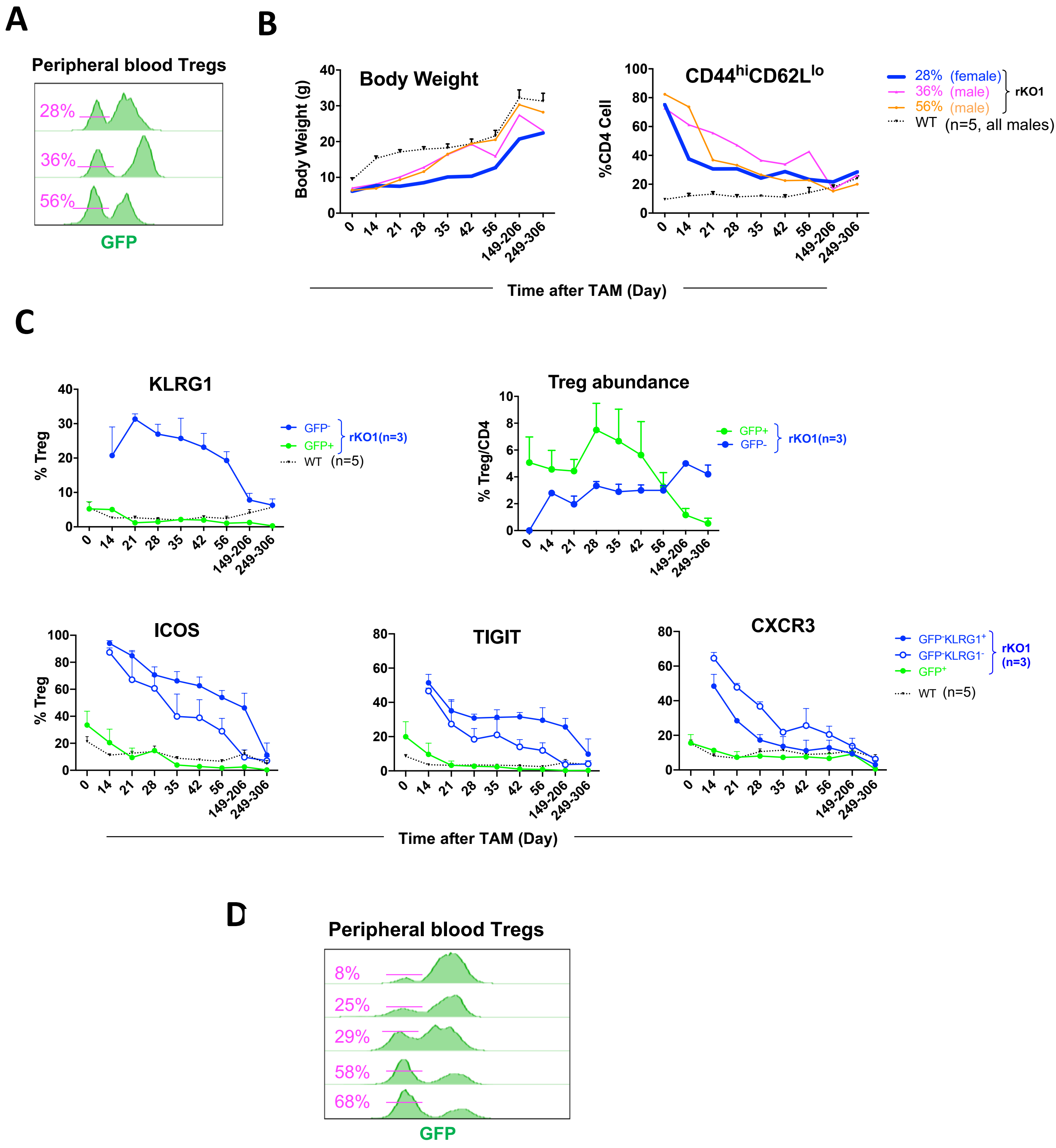


**Supplementary Figure 2. rKO1 and rKO2 mice rescued by TAM (low dose)**

(A-C) The three rKO1 mice. Shown are their KO reversal rates (A), body weight and blood E/M CD4 cells (B) and SuperTreg fate (C). The mouse with least *Brg1* re-expressed Tregs (28%) is highlighted with thick blue line. As the rKO mice (bearing 5-6 alleles) were quite rare, the 3 mice were born and hence analyzed at different times, except for the last two time points when the mice from different litters were analyzed together. The control mice were the same as those used for the rKO2 mice (Fig. 2H; Fig. 6). One of the rKO1 mice got very sick after TAM injection, and so we did not collect the blood for FACS on Day 14 and 21. (D) The KO reversal rates of the 5 rKO2 mice described in Fig. 2F, Fig. 2H and Fig. 6

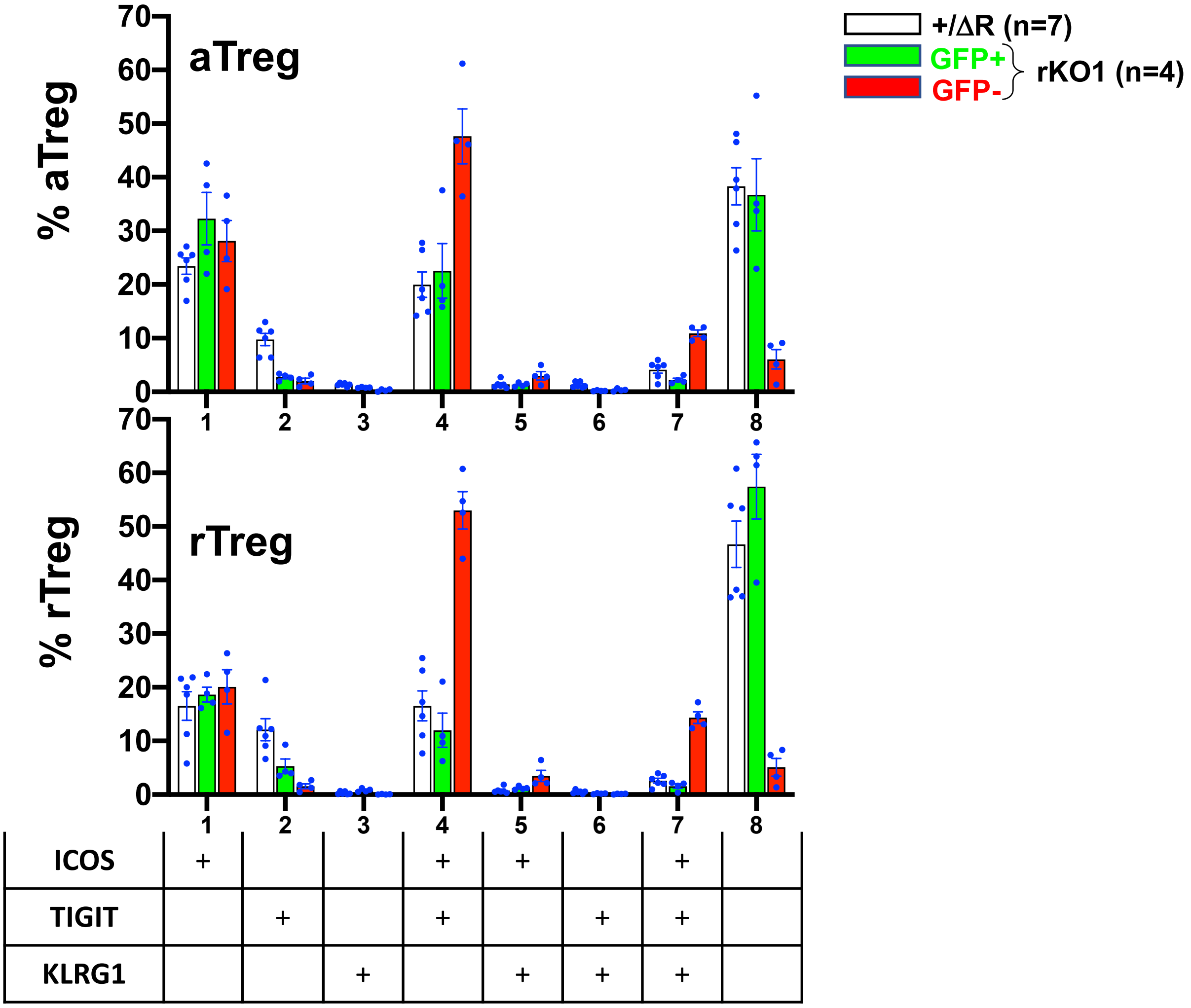


| | 1 | 2 | 3 | 4 | 5 | 6 | 7 | 8 |
|---|---|---|---|---|---|---|---|---|
| ICOS | + | | | + | + | | + | |
| TIGIT | | + | | + | | + | + | |
| KLRG1 | | | + | | + | + | + | |

**Supplementary Figure 3. Heterogeneity in marker expression**. Tregs could be classified into 8 subsets based on the expression of ICOS, TIGIT and KLRG1, the three markers co-stained in our assay. In both aTregs and rTregs, Brg1 reexpression increased Subset 4 (ICOS+TIGIT+) ,5 (ICOS+KLRG1+) and 7 (ICOS+TIGIT+KLRG1+) while depleting Subset 2 ($TIGIT^+$) without affecting Subset 1 ($ICOS^+$) and 6 ($KLRG1^+$ $TIGIT^+$). Overall, marker expression was greatly increased and so the cells lacking marker expression (Subset 8) severely depleted.

**A**

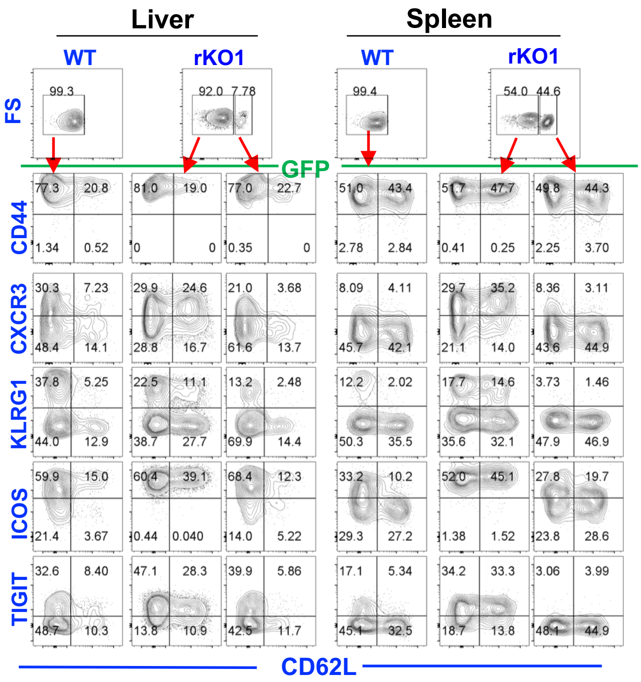


**B**

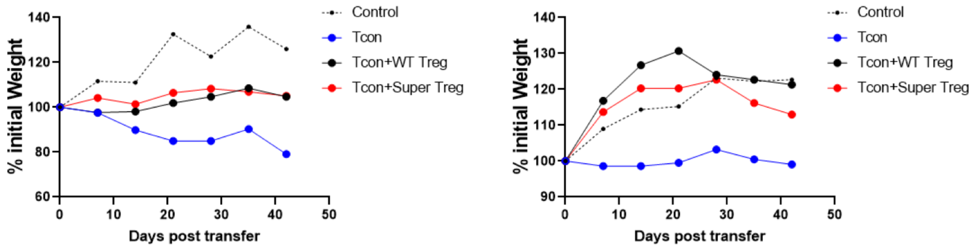


**Supplementary Figure 4. Treg function in vivo**

(A) Hepatic SuperTregs were hyperactivated as the splenic counterpart, although rTregs were relatively depleted in hepatic Treg pool presumably because CD62L retarded trafficking to inflamed tissues. (B) SuperTregs were of similar potency to WT Tregs in preventing weight loss in an IBD model. 6wks-old Rag2 KO males were adoptively transferred with 0.4 million naïve CD4 cells from B6 mice alone or in combination with 0.25 million Tregs, and body weight monitored thereafter. Shown are two independent experiments (n=1 in each case).

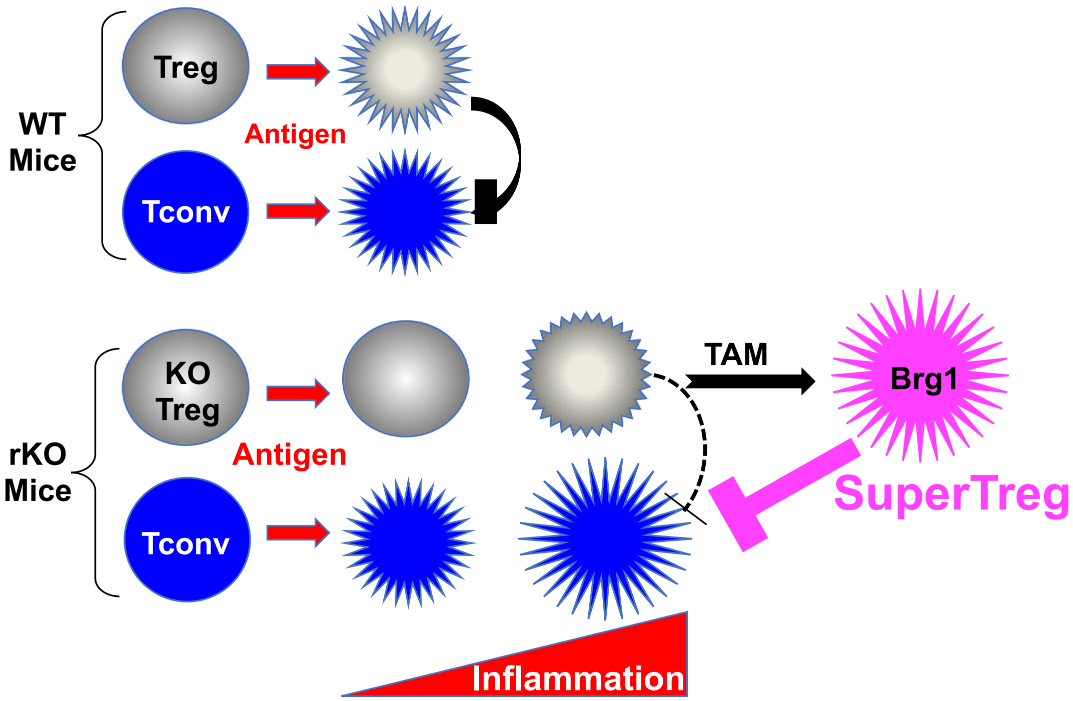


**Supplementary Figure 5. A model.** See text for detail.

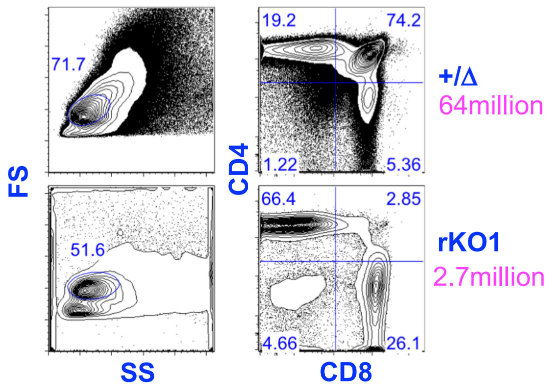


**Supplementary Figure 6. Thymocytes in 3-wk-old rKO1 mouse.** Cells were stained with CD4 and CD8 antibodies before analysis. DP was virtually absent, consistent with a dramatic decrease in thymocyte number (from 64 million to 2.7 million).
